# Microbially-derived short-chain fatty acids impact astrocyte gene expression in a sex-specific manner

**DOI:** 10.1016/j.bbih.2021.100318

**Published:** 2021-08-06

**Authors:** Simon Spichak, Francisco Donoso, Gerard M. Moloney, Eoin Gunnigle, Jillian M. Brown, Martin Codagnone, Timothy G. Dinan, John F. Cryan

**Affiliations:** a Department of Anatomy and Neuroscience, University College Cork, Cork, Ireland; b APC Microbiome Institute, University College Cork, Cork, Ireland; c Department of Psychiatry and Neurobehavioural Science, University College Cork, Cork, Ireland

**Keywords:** Short-chain fatty acid, Astrocyte, Microbiome, Neuro-immunity, Glia

## Abstract

Recent investigations in neuroscience implicate the role of microbial-derived metabolites, such as short-chain fatty acids (SCFAs) in brain health and disease. The SCFAs acetate, propionate and butyrate have pleiotropic effects within the nervous system. They are crucial for the maturation of the brain's innate immune cells, the microglia, and modulate other glial cells through the aryl-hydrocarbon receptor. Investigations in preclinical and clinical models find that SCFAs exert neuroprotective and antidepressant affects, while also modulating the stress response and satiety*.* However, many investigations thus far have not assessed the impact of sex on SCFA activity. Our novel investigation tested the impact of physiologically relevant doses of SCFAs on male and female primary cortical astrocytes. We find that butyrate (0–25 ​μM) correlates with increased *Bdnf* and *Pgc1-α* expression, implicating histone-deacetylase inhibitor pathways. Intriguingly, this effect is only seen in females. We also find that acetate (0–1500 ​μM) correlates with increased *Ahr* and *Gfap* expression in males only, suggesting immune modulatory pathways. In males, propionate (0–35 ​μM) correlates with increased *Il-22* expression, further suggesting immunomodulatory actions. These findings show a novel sex-dependent impact of acetate and butyrate, but not propionate on astrocyte gene expression.

## 1 Introduction

The microbiota-gut-brain axis emerged in recent years as a contributor or mediator of many neurophysiological and behavioural processes. The trillions of microorganisms within the mammalian gut, collectively called the microbiota, communicate with the brain through neuroendocrine, immune or vagal signalling (Cryan et al., 2019). They generate a multitude of neuroactive metabolites, absorbed into peripheral circulation, however it is unclear if these metabolites act on brain cells directly. By analyzing the predicted functions of gut bacteria, a recent study found pathways termed gut-brain modules, associated with quality of life and depression in a large human study (Valles-Colomer et al., 2019). Short-chain fatty acid (SCFA) producing bacteria, *Faecalibacteirum* and *Coprococcus* associated with quality of life, while *Coprococcus* was depleted in depression (Valles-Colomer et al., 2019). Thus, SCFAs are promising microbial-derived metabolites for modulating brain function.

SCFAs exert pleiotropic effects on cells through multiple pathways: immune signalling, as substrates in the Kreb's cycle, altering metabolic flux, histone deacetylase inhibition (HDACi), G protein-coupled receptor activation as well as other extracellular and intracellular receptors (Dalile et al., 2019). In preclinical and clinical studies, SCFAs are associated with neuroprotection (Sadler et al., 2020; Lee et al., 2020), stress-reduction (Yamawaki et al., 2018; Dalile et al., 2020), neuro-immunity (Correa-Oliveira et al., 2016), glutamate/glutamine metabolism (Frost et al., 2014), and appetite regulation (Frost et al., 2014). The effects of SCFAs are also mediated by sex (Shastri et al., 2015; Thion et al., 2018). Astrocytes are involved in many of these pathways in the brain (Belanger et al., 2011; Jensen et al., 2013; Alberini et al., 2018), and may mediate the effects of SCFAs. Importantly, astrocyte physiology and function differs greatly between the sexes (Arias et al., 2009; Hsu et al., 2001; Johnson et al., 2008; Santos-Galindo et al., 2011; Chistyakov et al., 2018).

Few studies have thus assessed the effects of SCFAs on naïve primary rodent or human astrocytes (Soliman et al., 2013; Wu et al., 2008; Yang et al., 2018; Kanski et al., 2014; Nuutinen et al., 2010; Garcez et al., 2020). To our knowledge, this is the first study examining the sex-specific effects of a physiologically relevant concentration of SCFAs on naïve primary mouse astrocytes. In this paper, we focused on gene expression changes across immune, metabolic and HDACi pathways.

## 2 Materials and methods

### 2.1 Animals

Experiments are authorised under a Euthanasia Only Project (2019-009). Experiments were conducted in accordance with guidelines established by established by University College Cork's Animal Ethical Experimentation Committee (AEEC) at University College Cork.

### 2.2 Seeding primary glial culture

Litters of C57BL/6 mice were sexed and euthanized between postnatal day 1–3. Three male pups and three female pups were pooled to control for the litter effect. The mouse brains were removed from the skulls and the cortices were dissected out, while also removing the meninges and hippocampus. They were pooled by sex into collection tubes, filled with DMEM-F12 (Gibco: 11320033) on ice. Pooled brains were washed with PBS 10 ​mM, then incubated with 1.5 ​mL of Trypsin/EDTA (0.25%) (Thermofisher: 25200072) at 37 ​°C, 5% CO_2_ for 20 ​min. Trypsin/EDTA (0.25%) was inactivated with 9 ​mL of DMEM/F12 with 10% heat inactivated Fetal Bovine Serum (Thermofisher: 16140071). This mixture was mechanically dissociated, transferred to a 15 ​mL Falcon Tube and centrifuged at 200 ​*g*, 21 ​°C for 10 ​min and the supernatant was removed and resuspended in media. Media was composed of DMEM (Gibco: 16219961) with 10% heat inactivated Fetal Bovine Serum, 1% Penicillin/Streptomycin (Gibco: 15070063) and 2 ​mM L-Glutamine (Thermofisher: 25030149) Cells were then seeded into T75 flasks with filtered caps, coated overnight with Poly-D-Lysine (Sigma-Aldrich: P6407-5 ​MG). Half of the cell media was removed every 3–4 days and replenished with new media, cells were grown at 37 ​°C, 5% CO_2_.

### 2.3 Primary astrocyte enrichment

After 14 days, cells were placed on an orbital shaker for 24 ​h at 150 ​rpm, 37 ​°C, 5% CO_2_. This process removed contaminating microglia from the culture. Media was aspirated, cells were washed with PBS 10 ​mM and media was replenished. Cells were than shaken for 24 ​h at 230 ​rpm, 37 ​°C, 5% CO_2_ to remove oligodendrocyte precursor cells. Cells were washed with PBS 10 ​mM and left for 1–2 days. Afterwards, astrocytes were dissociated using 3.0 ​mL Trypsin/EDTA (0.25%) before being seeded at a density of ~150 ​000 ​cells into a 6-well cell culture plate. Each well contained 1.5 ​mL of media and 150 ​000 ​cells.

### 2.4 Primary astrocyte purity

To confirm enrichment, cells from three different litters were grown on coverslips and fixed with 4% PFA (Sigma-Aldrich: 158127) in triplicate. Briefly cells were first blocked for an hour with a solution of PBS 10 ​mM with 0.1% Triton-X (Sigma-Aldrich X100) and 5% Donkey Serum (Sigma-Aldrich D9663). The primary antibodies, Rat anti-GFAP (1:250) (Thermofisher:13-0300) and Rabbit-anti IBA1 (1:1000) (Wako: 019-19741) were added in a PBS/0.1% Triton-X/2% Donkey Serum solution and left at 4 ​°C for 12 ​h. After three washes, a secondary antibody, anti-Rat Alexa Fluor 488 (Thermofisher: A-21028) and anti-Rabbit Alexa Fluor 594 (Thermofisher: A-11012) were both added in a 1:500 dilution of PBS/0.1% Triton-X/2% Donkey Serum for 2 ​h in a humid chamber. Cells were washed three times and DAPI (Thermofisher: D1306) was added at a concentration of 1:1000 in a PBS solution with 2% Donkey Serum. Finally, cells were mounted onto slides and GFAP/DAPI double positive cells were counted as astrocytes while DAPI positive cells or IBA1/DAPI double positive cells were counted as non-astrocytes. We found our enriched culture was >95% pure. In addition, T75 flasks were viewed under a microscope to ensure no microglia (small, rounded cells adherent to the surface layer of attached cells) were present before further experiments.

### 2.5 Cell culture treatments

After 5 days, once the cells adhered, 150 ​mL of media was removed, and new media with different concentrations of the SCFAs; acetate (Sigma-Aldrich: S7545), propionate (Sigma-Aldrich: P1880) or butyrate (Sigma-Aldrich, 303410) diluted in culture media were added. Physiologically relevant concentrations were estimated based on previous studies (Guardia-Escote et al., 2019). For acetate we tested concentrations at 150 ​μM, 750 ​μM and 1500 ​μM. For propionate we tested concentrations at 3.5 ​μM, 17.5 ​μM and 35 ​μM. For butyrate we tested concentrations at 2.5 ​μM, 12.5 ​μM and 25 ​μM.

### 2.6 Quantitative reverse transcription polymerase chain reaction (PCR)

24 ​h after treatment, cells were lysed and isolated according to the TRIzol protocol (Thermofisher: 15596026). RNA concentrations were then measured using a Nanodrop-1000 (Thermofisher) followed by the generation of cDNA using the High-Capacity cDNA Reverse Transcription Kit (Thermofisher: 4368814). Using the primers listed in Table 1 (Eurofins) along with the SYBR™ Green PCR Master Mix kit (Thermofisher: 4309155), we conducted a quantitative PCR in a Lightcycler 480 II (Roche).

**Table 1:** PCR primers.

Pathway	Gene	Sequence Forward (5’ -> 3’)	Sequence Reverse (5’ -> 3’)	Reference
**Astrocyte Marker**	GFAP	GCTCCAAGATGAAACCA ACC	TTCAACCTTTCTCTCCAA ATCC	(Zeisel et al., 2018; Ramos-Garcia et al., 2020)
**Aryl-Hydrocarbon Receptor Pathway**	Aryl-hydrocarbon receptor	ACGGATGAAGAAGGACG AG	AAGGAGGACACAGATAG ATGG	(Ramos-Garcia et al., 2020; Rothhammer et al., 2016)
	S100β	TCTGTCTACACTCCTGTT ACTC	TCTCCATCACTTTGTCCA CC	Tomova et al. (2019)
	IL-22	TGACGACCAGAACATCC AG	TAGAAGGCAGGAAGGAG CAG	Monteleone et al. (2011)
	IFNAR1	TCTCAAAAACACATTCTC CCTC	CCATCCTTCTCCATGCTT ATC	Rothhammer et al. (2016)
	CYP1B1	AAGGAAGGGGAGTGCG ATAG	AATAGATGGGGGAGATA GGAGG	Rothhammer et al. (2016)
**Glutamate-Glutamine Cycle**	GLUL	TCTCTACACACCAACCC TTTC	ACCAACCTTCAACTCCT CAC	Schousboe et al. (2014)
	GAD67	TGTGAGCCAAAGAGAAA AGATG	TGAGGGGGGAAAGAGA AGAG	Schousboe et al. (2014)
	GLUD1	CTTCTTTACCACCTCTTC ACC	ACCTAAAAGCAAACCAC CTAAC	Schousboe et al. (2014)
**HDAC Inhibition Pathway**	GDNF	TTCAACTCTTTTTCCCCC TTC	TTCCCCTATGTTCTCCTG TC	Bourassa et al. (2016)
	BDNF	TTCCCCTATGTTCTCCTG TC	TACCATTCCCCACCTCC ATC	Bourassa et al. (2016)
	NGF1	AGCAAAGCCAAGCAAAC C	CAAAACCCAACCAAACA AACC	Bourassa et al. (2016)
	SP1	ATGCTGCTCAACTCTCC TC	GCTATTCTCTCCTTCTCC ACC	Bourassa et al. (2016)
	PGC1-α	AACTCCTCCCACAACTC CTC	GCCGTTTAGTCTTCCTTT CC	Bourassa et al. (2016)

### 2.7 Gene expression analysis and statistics

Each sample was analysed in duplicate for both target gene and reference gene (β-actin), and the relative mRNA expressions were calculated using the 2^−ΔΔCt^ method. Linear regression was used to test for SCFA-dose responses (Livak and Schmittgen, 2001). Results in bar plots are presented as mean ​± ​SEM. For presentation in heat maps, the means for gene expression were scaled. For linear regression, the SCFA dose was treated as a continuous independent variable while gene expression was treated as a continuous dependent variable. Graphs were made using ggplot2 in R 4.0.0. Linear regression was performed using the lm function in R 4.0.0. The code used for analysis and plotting is available here.

## 3 Results

### 3.1 Butyrate impacts *Bdnf* and *Pgc1-α* expression in females only

**Fig. 1:**
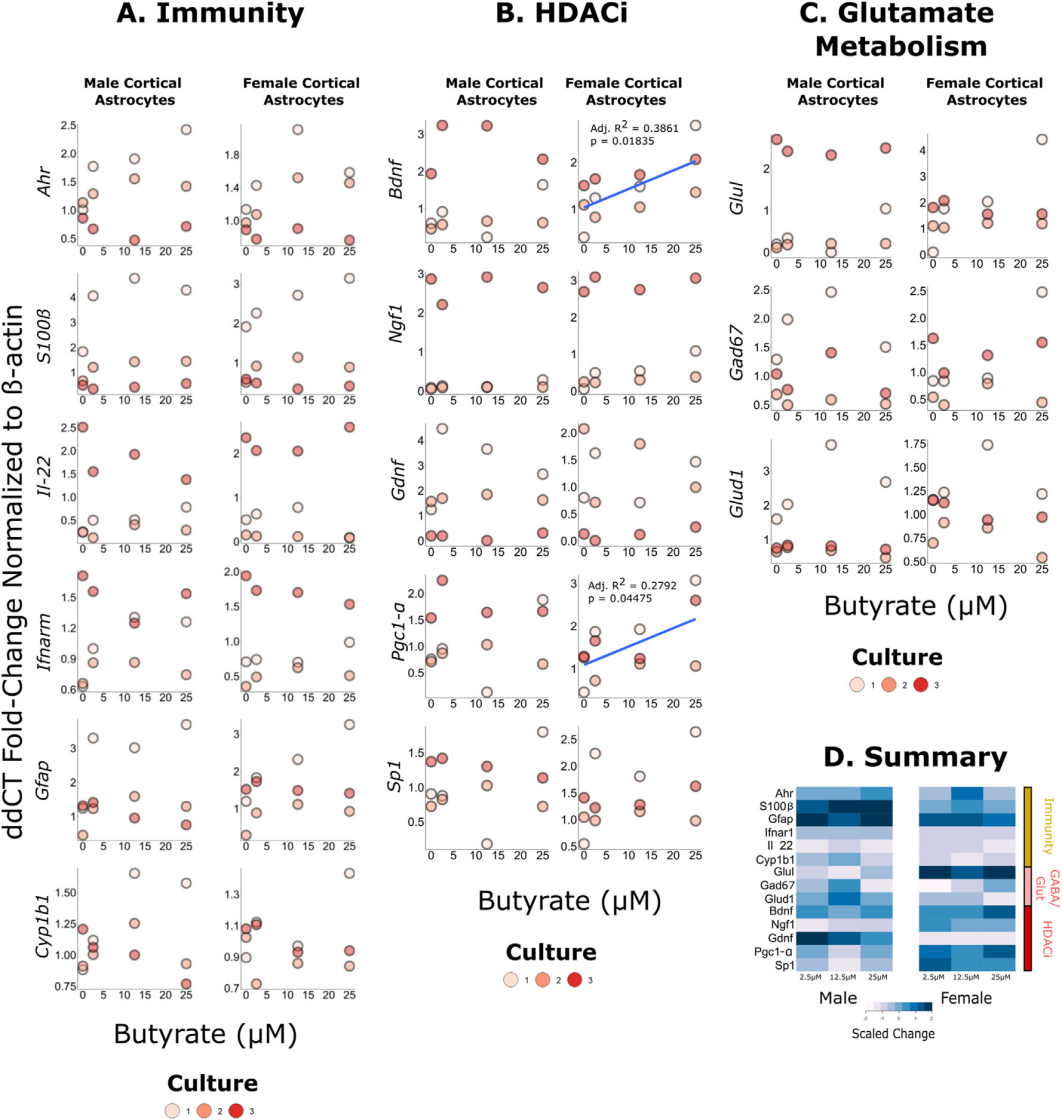

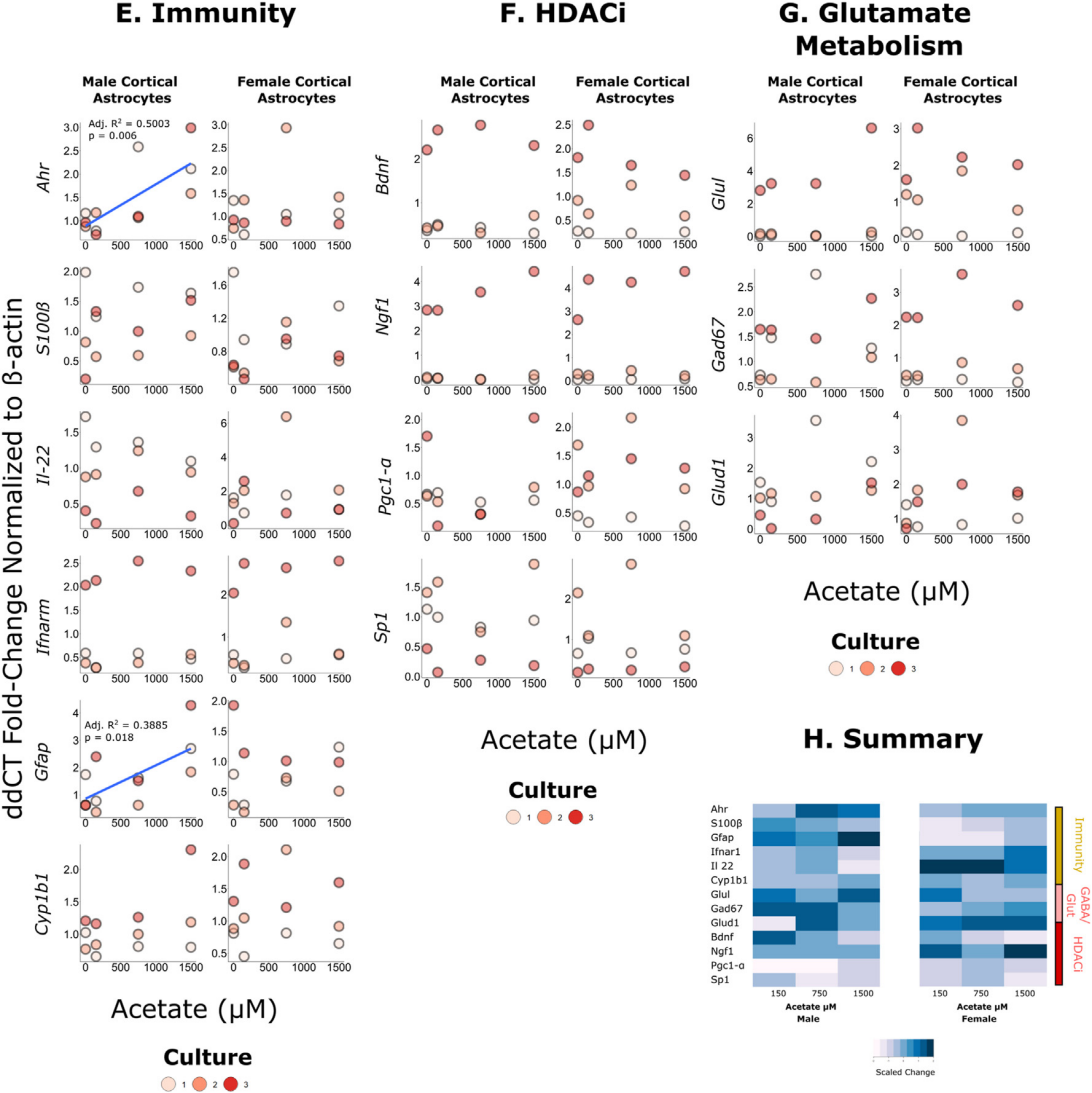

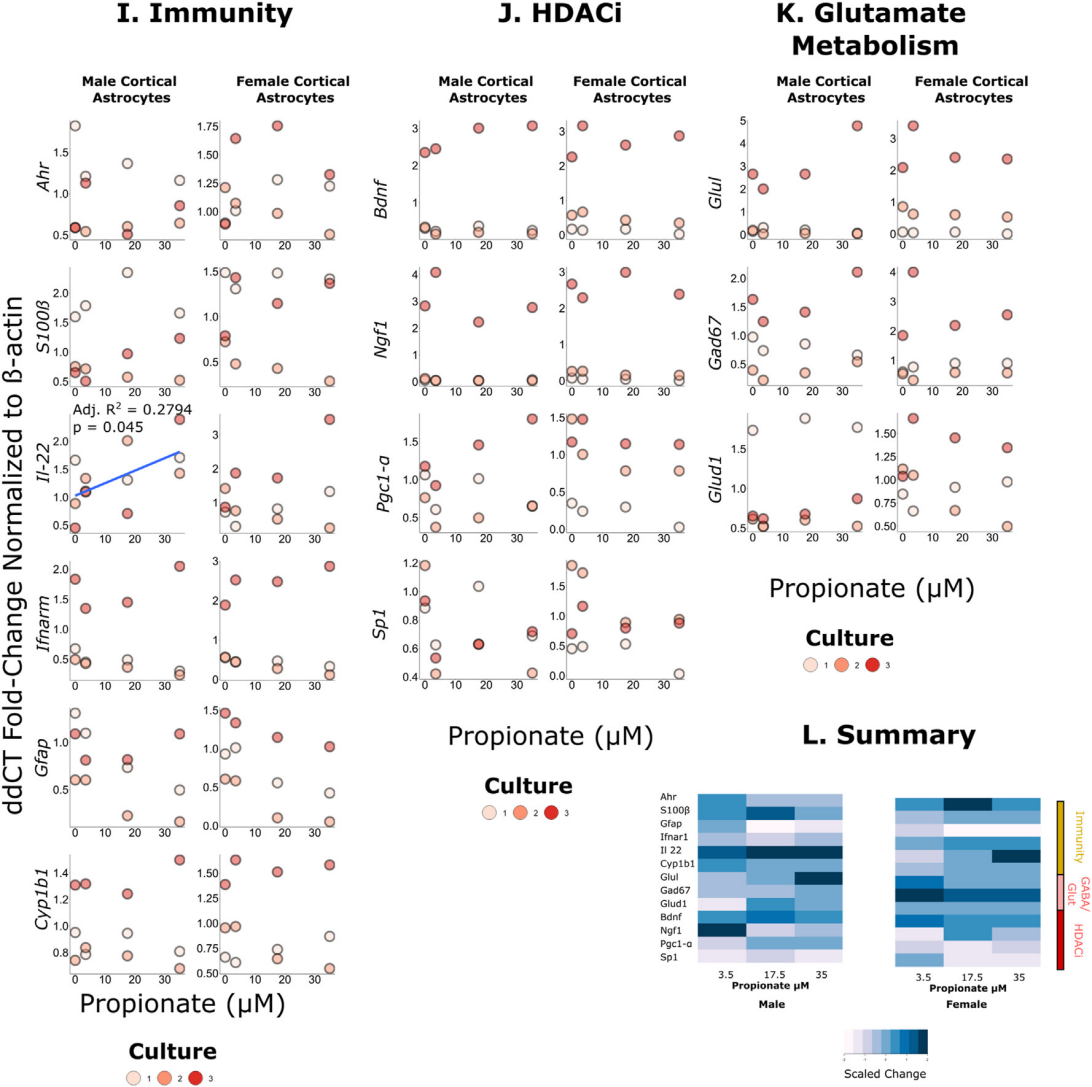
Impact of SCFAs on male and female cortical astrocyte gene expression (N ​= ​3 litters). Raw data was analysed using the 2^-ΔΔCT^ method normalized to the control. Associations were analysed using linear regression. *A-D.* The impact of different concentrations of butyrate on immune-related, glutamine/GABA metabolism and histone deacetylase inhibitory pathways. There is a significant association between butyrate dose and *Bdnf* expression in female cortical astrocytes (df = 10, residual standard error = 0.9446, R^2^ = 0.4419, Adjusted R^2^ = 0.3861, F-Statistic = 7.918, p = 0.01835) but not in males (df = 10, residual standard error = 1.386, R^2^ = 0.01261, Adjusted R^2^ = −0.08612, F-Statistic = 0.1278, p = 0.7282). There is a significant association between butyrate dose and *Pgc1-α* in female cortical astrocytes (df = 10, residual standard error = 0.9229, R^2^ = 0.3447, Adjusted R^2^ = 0.2792, F-Statistic = 5.26, p = 0.04475) but not in males (df = 10, residual standard error = 0.6803, R^2^ = 0.1897, Adjusted R^2^ = −0.07913, F-Statistic = 0.1934, p = 0.6995). *D.* A heatmap visualizing mean changes in gene expression across different doses of butyrate. *E-H.* The impact of different concentrations of acetate on immune-related, glutamine/GABA metabolism and histone deacetylase inhibitory pathways. There is a significant association between acetate dose and *Ahr* expression in male cortical astrocytes (df = 10, residual standard error = 0.5271, R^2^ = 0.5457, Adjusted R^2^ = 0.5003, F-Statistic = 12.01, p = 0.00606) but not in females (df = 10, residual standard error = 0.6358, R^2^ = 0.0243, Adjusted R^2^ = −0.07327, F-Statistic = 0.2491, p = 0.6285). There is a significant association between acetate dose and *Gfap* in male cortical astrocytes (df = 10, residual standard error = 0.8891, R^2^ = 0.4414, Adjusted R^2^ = 0.3855, F-Statistic = 7.902, p = 0.01844) but not in females (df = 10, residual standard error = 0.5173, R^2^ = 0.008557, Adjusted R^2^ = −0.09059, F-Statistic = 0.0863, p = 0.7749). *H*. A heatmap visualizing mean changes in gene expression across different genes. *I-L.* The impact of different concentrations of acetate on immune-related, glutamine/GABA metabolism and histone deacetylase inhibitory pathways. There is a significant association between propionate dose and *IL-22* expression in male cortical astrocytes (df = 10, residual standard error = 0.4661, R^2^ = 0.345, Adjusted R^2^ = 0.279, F-Statistic = 5.266, p = 0.045) but not in females (df = 10, residual standard error = 0.8874, R^2^ = 0.0955, Adjusted R^2^ = 0.005231, F-Statistic = 1.058, p = 0.3279). *K*. A heatmap visualizing mean changes in gene expression across different genes.

Low levels of butyrate (2.5–25 ​μM) did not significantly impact gene expression in male astrocyte cultures (see Fig. 1). However, in female astrocytes, *Bdnf* expression positively associated with butyrate dose (df ​= ​10, residual standard error ​= ​0.9446, R^2^ ​= ​0.4419, Adjusted R^2^ ​= ​0.3861, F-Statistic ​= ​7.918, p ​= ​0.01835). Similarly, the expression of *Pgc1-α* was positively associated with butyrate dose in female astrocytes (df ​= ​10, residual standard error ​= ​0.9229, R^2^ ​= ​0.3447, Adjusted R^2^ ​= ​0.2792, F-Statistic ​= ​5.26, p ​= ​0.04475) but not in male astrocytes (see Supplemental Tables 1–2).

### 3.2 Acetate impacts *Ahr* and *Gfap* expression in male astrocyte cultures only

Low levels of acetate (2.5–25 ​μM) did not significantly impact gene expression in female astrocytes (see Fig. 1). However, in males *Ahr* expression positively associated with acetate dose (df ​= ​10, residual standard error ​= ​0.5271, R^2^ ​= ​0.5457, Adjusted R^2^ ​= ​0.5003, F-Statistic ​= ​12.01, p ​= ​0.00606). The expression of *Gfap* was positively associated with acetate dose in male astrocytes (df ​= ​10, residual standard error ​= ​0.8891, R^2^ ​= ​0.4414, Adjusted R^2^ ​= ​0.3855, F-Statistic ​= ​7.902, p ​= ​0.01844) but not in female astrocytes (see Supplemental Tables 3–4).

### 3.3 Propionate impacts *IL-22* gene expression in male astrocyte cultures only

Propionate treatment (3.5–35 ​μM) increased gene expression of *IL-*22 in male astrocyte cultures only (df ​= ​10, residual standard error ​= ​0.4661, R^2^ ​= ​0.345, Adjusted R^2^ ​= ​0.279, F-Statistic ​= ​5.266, p ​= ​0.045) (see Fig. 1 and Supplemental Tables 5–6).

## 4 Discussion

To our knowledge, this is the first *in vitro* study that assessed the sex-specific impact of physiologically relevant levels of SCFAs in cortical astrocytes. We used primary enriched male and female cortical astrocyte cultures to investigate the impacts of butyrate, acetate and propionate on gene expression across aryl-hydrocarbon receptor/immune signalling, glutamate/glutamine metabolism and histone deacetylase inhibitor HDACi pathways.

In female, but not male, astrocyte cultures we found that butyrate concentrations positively correlated with *Bdnf* and *Pgc1-α* expression. These may be activated downstream of HDACi activity. Previous rodent studies found environmental enrichment increased *Bdnf* expression more in female mice than male mice (Zhu et al., 2006). Here, butyrate may be activating the same pathways as environmental enrichment, a result of female cells being more responsive to neurogenic stimuli.

In male, but not female cortical astrocytes, acetate treatment positively correlated with *Ahr* and *Gfap* expression, suggesting acetate is involved in the anti-inflammatory pathway.

Propionate only increased the expression of *Il-22* significantly in males. In the gut, this cytokine exerts anti-inflammatory properties after Aryl-hydrocarbon receptor in the gut (Monteleone et al., 2011). In brain astrocytes, *Il-22* expression promotes cell survival (Perriard et al., 2015). Females are more likely to develop neuroinflammatory disorders than males (Gold et al., 2019). This sex-specific activation may reflect a neuroprotective pathway specific to males.

This may stimulate a defensive pathway When released from Th17 ​cells in the periphery, *Il-22* may drive immunopathogenesis in multiple sclerosis, and may mediate anti-inflammatory activity in astrocytes through a similar pathway.

It is unclear why only a few genes in a specific pathway were upregulated or downregulated in our study. However, it underlies the importance of accounting for litter and sex effects going forward.

There are practical limitations within the study. Prior to this investigation, there was little insight into pathways perturbed by small concentrations of SCFAs; other pathways we didn't test may have been affected. Changes in gene expression do not always translate into changes in protein. Further, many of the associations were low-to-moderate according R^2^ adjusted metrics.

We also found large levels of variance across independent cultures. While the expression of some genes varied greatly between the cultures, others did not (see Supplemental Figs. 1–3, Supplemental Tables 7–9). Using three different animals from the same litter lowers variance but results in pseudo-replication. A larger sample size of litters would reduce the variance.

In summary, we present evidence that mammalian astrocytes may mediate some of these effects in a sex-specific manner. This adds to the existing literature suggesting the importance of astrocytes in brain health and disease, and among the few studies assessing astrocyte function in the context of the microbiome.

## Funding sources

The 10.13039/501100014745APC Microbiome Institute is a research institute funded by 10.13039/501100001602Science Foundation Ireland (10.13039/501100001602SFI) through the Irish Government's National Development Plan. J.F.C., T.G.D. and S.S. are supported by 10.13039/501100001602SFI (Grant Nos. 10.13039/501100001602SFI/12/RC/2273_P2). S.S. is also funded through the 10.13039/501100002081Irish Research Council (GOIPG/2018/2560).

## Declaration of competing interest

J.F.C and T.G.D have research support from Cremo, Pharmavite, Dupont and Nutricia. These authors have spoken at meetings sponsored by food and pharmaceutical companies. All other authors report no potential conflicts of interests.

## References

[bib1] Alberini C.M. (2018). Astrocyte glycogen and lactate: new insights into learning and memory mechanisms. Glia.

[bib2] Arias C. (2009). Sex and estrous cycle-dependent differences in glial fibrillary acidic protein immunoreactivity in the adult rat hippocampus. Horm. Behav..

[bib3] Belanger M., Allaman I., Magistretti P.J. (2011). Brain energy metabolism: focus on astrocyte-neuron metabolic cooperation. Cell Metabol..

[bib4] Bourassa M.W. (2016). Butyrate, neuroepigenetics and the gut microbiome: can a high fiber diet improve brain health?. Neurosci. Lett..

[bib5] Chistyakov D.V. (2018). Sex-mediated differences in LPS induced alterations of TNFα, IL-10 expression, and prostaglandin synthesis in primary astrocytes. Int. J. Mol. Sci..

[bib6] Correa-Oliveira R. (2016). Regulation of immune cell function by short-chain fatty acids. Clin Transl Immunology.

[bib7] Cryan J.F. (2019). The microbiota-gut-brain Axis. Physiol. Rev..

[bib8] Dalile B. (2019). The role of short-chain fatty acids in microbiota-gut-brain communication. Nat. Rev. Gastroenterol. Hepatol..

[bib9] Dalile B. (2020). Colon-delivered short-chain fatty acids attenuate the cortisol response to psychosocial stress in healthy men: a randomized, placebo-controlled trial. Neuropsychopharmacology.

[bib10] Frost G. (2014). The short-chain fatty acid acetate reduces appetite via a central homeostatic mechanism. Nat. Commun..

[bib11] Garcez M.L. (2020). Sodium butyrate and indole-3-propionic acid prevent the increase of cytokines and kynurenine levels in LPS-induced human primary astrocytes. Int. J. Tryptophan Res..

[bib12] Gold S.M. (2019). Sex differences in autoimmune disorders of the central nervous system. Semin. Immunopathol..

[bib13] Guardia-Escote L. (2019). APOE genotype and postnatal chlorpyrifos exposure modulate gut microbiota and cerebral short-chain fatty acids in preweaning mice. Food Chem. Toxicol..

[bib14] Hsu C. (2001). Sexually dimorphic effect of glutamate treatment on cell cycle arrestment of astrocytes from the preoptic area of neonatal rats. Dev. Neurosci..

[bib15] Jensen C.J., Massie A., De Keyser J. (2013). Immune players in the CNS: the astrocyte. J. Neuroimmune Pharmacol..

[bib16] Johnson R.T., Breedlove S.M., Jordan C.L. (2008). Sex differences and laterality in astrocyte number and complexity in the adult rat medial amygdala. J. Comp. Neurol..

[bib17] Kanski R. (2014). Histone acetylation in astrocytes suppresses GFAP and stimulates a reorganization of the intermediate filament network. J. Cell Sci..

[bib18] Lee J. (2020). Gut microbiota-derived short-chain fatty acids promote post-stroke recovery in aged mice. Circ. Res..

[bib19] Livak K.J., Schmittgen T.D. (2001). Analysis of relative gene expression data using real-time quantitative PCR and the 2(-Delta Delta C(T)) Method. Methods.

[bib20] Monteleone I. (2011). Aryl hydrocarbon receptor-induced signals up-regulate IL-22 production and inhibit inflammation in the gastrointestinal tract. Gastroenterology.

[bib21] Nuutinen T. (2010). Valproic acid stimulates clusterin expression in human astrocytes: implications for Alzheimer's disease. Neurosci. Lett..

[bib22] Perriard G. (2015). Interleukin-22 is increased in multiple sclerosis patients and targets astrocytes. J. Neuroinflammation.

[bib23] Ramos-Garcia N.A. (2020). Aryl hydrocarbon receptor in post-mortem Hippocampus and in Serum from young, elder, and alzheimer's patients. Int. J. Mol. Sci..

[bib24] Rothhammer V. (2016). Type I interferons and microbial metabolites of tryptophan modulate astrocyte activity and central nervous system inflammation via the aryl hydrocarbon receptor. Nat. Med..

[bib25] Sadler R. (2020). Short-chain fatty acids improve poststroke recovery via immunological mechanisms. J. Neurosci..

[bib26] Santos-Galindo M. (2011). Sex differences in the inflammatory response of primary astrocytes to lipopolysaccharide. Biol. Sex Differ..

[bib27] Schousboe A. (2014). Glutamate metabolism in the brain focusing on astrocytes. Adv Neurobiol.

[bib28] Shastri P. (2015). Sex differences in gut fermentation and immune parameters in rats fed an oligofructose-supplemented diet. Biol. Sex Differ..

[bib29] Soliman M.L., Combs C.K., Rosenberger T.A. (2013). Modulation of inflammatory cytokines and mitogen-activated protein kinases by acetate in primary astrocytes. J. Neuroimmune Pharmacol..

[bib30] Thion M.S. (2018). Microbiome influences prenatal and adult microglia in a sex-specific manner. Cell.

[bib31] Tomova A. (2019). Specificity of gut microbiota in children with autism spectrum disorder in Slovakia and its correlation with astrocytes activity marker and specific behavioural patterns. Physiol. Behav..

[bib32] Valles-Colomer M. (2019). The neuroactive potential of the human gut microbiota in quality of life and depression. Nat Microbiol.

[bib33] Wu X. (2008). Histone deacetylase inhibitors up-regulate astrocyte GDNF and BDNF gene transcription and protect dopaminergic neurons. Int. J. Neuropsychopharmacol..

[bib34] Yamawaki Y. (2018). Sodium butyrate abolishes lipopolysaccharide-induced depression-like behaviors and hippocampal microglial activation in mice. Brain Res..

[bib35] Yang T. (2018). Butyrate regulates inflammatory cytokine expression without affecting oxidative respiration in primary astrocytes from spontaneously hypertensive rats. Phys. Rep..

[bib36] Zeisel A. (2018). Molecular architecture of the mouse nervous system. Cell.

[bib37] Zhu S.W. (2006). Influence of differential housing on emotional behaviour and neurotrophin levels in mice. Behav. Brain Res..

