# Supplementary material for: Microbially-derived short-chain fatty acids impact astrocyte gene expression in a sex-specific manner
Source: Brain Behav Immun Health. 2021 Aug 6;16:100318. doi: 10.1016/j.bbih.2021.100318 (PMC8474187; doi:10.1016/j.bbih.2021.100318)
Supplement: Multimedia component 1 [file mmc1.docx]

**Supplemental Table 1.** Male astrocyte cultures treated with butyrate (linear regression)

| **Gene** | **Estimates for Dose** | ***p-*Value** | **R/R^2^ adjusted** |
| --- | --- | --- | --- |
| *Ahr* | 0.02 (-0.02 – 0.05) | 0.328 | 0.096/0.005 |
| *S100β* | 0.03 (-0.08 – 0.14) | 0.510 | 0.045/-0.051 |
| *Gfap* | 0.02 (-0.05 – 0.09) | 0.503 | 0.046/-0.049 |
| *Ifnar* | 0.01 (-0.02 – 0.03) | 0.657 | 0.021/-0.077 |
| *Il-22* | -0.00 (-0.06 – 0.05) | 0.929 | 0.001/-0.099 |
| *Cyp1b1* | 0.00 (-0.01 – 0.02) | 0.612 | 0.027/-0.071 |
| *Glul* | 0.01 (-0.07 – 0.08) | 0.798 | 0.007/-0.092 |
| *Bdnf* | 0.01 (-0.06 – 0.09) | 0.728 | 0.013/-0.086 |
| *Ngf1* | 0.00 (-0.08 – 0.09) | 0.914 | 0.001/-0.099 |
| *Gdnf* | 0.00 (-0.09 – 0.10) | 0.924 | 0.001/-0.099 |
| *Gad67* | -0.00 (-0.05 – 0.04) | 0.915 | 0.001/-0.099 |
| *Glud1* | 0.01 (-0.06 – 0.08) | 0.709 | 0.015/-0.084 |
| *Pgc1-α* | 0.01 (-0.03 – 0.05) | 0.669 | 0.019/-0.079 |
| *Sp1* | 0.01 (-0.02 – 0.04) | 0.611 | 0.027/-0.070 |

**Supplemental Table 2.** Female astrocyte cultures treated with butyrate (linear regression)

| **Gene** | **Estimates for Dose** | ***p-*Value** | **R/R^2^ adjusted** |
| --- | --- | --- | --- |
| *Ahr* | 0.01 (-0.00 – 0.04) | 0.342 | 0.091/-0.000 |
| *S100β* | 0.02 (-0.05 – 0.08) | 0.582 | 0.031/-0.066 |
| *Gfap* | 0.03 (-0.02 – 0.08) | 0.219 | 0.147/0.062 |
| *Ifnar* | 0.00 (-0.04 – 0.04) | 0.971 | 0.000/-0.100 |
| *Il-22* | -0.00 (-0.07 – 0.07) | 0.953 | 0.000/-0.100 |
| *Cyp1b1* | 0.00 (-0.01 – 0.01) | 0.653 | 0.021/-0.077 |
| *Glul* | 0.05 (-0.02 – 0.11) | 0.135 | 0.209/0.130 |
| *Bdnf* | 0.04 (0.01 – 0.07) | **0.018** | 0.442/0.386 |
| *Ngf1* | 0.02 (-0.07 – 0.10) | 0.689 | 0.017/-0.082 |
| *Gdnf* | -0.00 (-0.05 – 0.05) | 0.989 | 0.000/-0.100 |
| *Gad67* | 0.02 (-0.01 – 0.06) | 0.183 | 0.170/0.087 |
| *Glud1* | -0.00 (-0.02 – 0.02) | 0.668 | 0.019/-0.079 |
| *Pgc1-α* | 0.04 (0.00 – 0.08) | **0.045** | 0.345/0.279 |
| *Sp1* | 0.02 (-0.01 – 0.06) | 0.207 | 0.154/0.069 |

**Supplemental Table 3.** Male astrocyte cultures treated with acetate (linear regression)

| **Gene** | **Estimates for Dose** | ***p-*Value** | **R/R^2^ adjusted** |
| --- | --- | --- | --- |
| *Ahr* | 0.00 (0.00 – 0.00) | **0.006** | 0.546/0.500 |
| *S100β* | 0.00 (-0.00 – 0.00) | 0.413 | 0.068/-0.025 |
| *Gfap* | 0.00 (0.00 – 0.00) | **0.018** | 0.441/0.386 |
| *Ifnar* | 0.00 (-0.00 – 0.00) | 0.786 | 0.008/-0.092 |
| *Il-22* | -0.00 (-0.00 – 0.00) | 0.787 | 0.008/-0.092 |
| *Cyp1b1* | 0.00 (-0.00 – 0.00) | 0.141 | 0.204/0.124 |
| *Glul* | 0.00 (-0.00 – 0.00) | 0.462 | 0.055/-0.039 |
| *Bdnf* | 0.00 (-0.00 – 0.00) | 0.971 | 0.000/-0.000 |
| *Ngf1* | 0.00 (-0.00 – 0.00) | 0.670 | 0.019/-0.079 |
| *Gad67* | 0.00 (-0.00 – 0.00) | 0.432 | 0.090/-0.001 |
| *Glud1* | 0.00 (-0.00 – 0.00) | 0.206 | 0.154/0.070 |
| *Pgc1-α* | 0.00 (-0.00 – 0.00) | 0.505 | 0.046/-0.050 |
| *Sp1* | -0.00 (-0.00 – 0.00) | 0.994 | 0.000/-0.100 |

**Supplemental Table 4.** Female astrocyte cultures treated with acetate (linear regression)

| **Gene** | **Estimates for Dose** | ***p-*Value** | **R/R^2^ adjusted** |
| --- | --- | --- | --- |
| *Ahr* | 0.00 (-0.00 – 0.00) | 0.629 | 0.024/-0.073 |
| *S100β* | 0.00 (-0.00 – 0.00) | 0.738 | 0.012/-0.087 |
| *Gfap* | 0.00 (-0.00 – 0.00) | 0.775 | 0.009/-0.091 |
| *Ifnar* | 0.00 (-0.00 – 0.00) | 0.674 | 0.018/-0.080 |
| *Il-22* | 0.00 (-0.00 – 0.00) | 0.855 | 0.003/-0.096 |
| *Cyp1b1* | 0.00 (-0.00 – 0.00) | 0.894 | 0.002/-0.098 |
| *Glul* | -0.00 (-0.00 – 0.00) | 0.853 | 0.004/-0.096 |
| *Bdnf* | -0.00 (-0.00 – 0.00) | 0.629 | 0.024/-0.073 |
| *Ngf1* | 0.00 (-0.00 – 0.00) | 0.755 | 0.010/-0.089 |
| *Gad67* | 0.00 (-0.00 – 0.00) | 0.772 | 0.009/-0.090 |
| *Glud1* | 0.00 (-0.00 – 0.00) | 0.458 | 0.056/-0.038 |
| *Pgc1-α* | -0.00 (-0.00 – 0.00) | 0.957 | 0.000/-0.100 |
| *Sp1* | -0.00 (-0.00 – 0.00) | 0.847 | 0.004/-0.096 |

**Supplemental Table 5.** Male astrocyte cultures treated with propionate (linear regression)

| **Gene** | **Estimates for Dose** | ***p-*Value** | **R/R^2^ adjusted** |
| --- | --- | --- | --- |
| *Ahr* | 0.00 (-0.02 – 0.02) | 0.716 | 0.014/-0.085 |
| *S100β* | 0.01 (-0.02 – 0.03) | 0.706 | 0.015/-0.084 |
| *Gfap* | -0.01 (-0.03 – 0.00) | 0.141 | 0.203/0.123 |
| *Ifnar* | -0.00 (-0.03 – 0.03) | 0.928 | 0.001/-0.099 |
| *Il-22* | 0.02 (0.00 – 0.04) | **0.045** | 0.345/0.279 |
| *Cyp1b1* | 0.00 (-0.01 – 0.01) | 0.990 | 0.000/-0.100 |
| *Glul* | 0.02 (-0.05 – 0.10) | 0.562 | 0.035/-0.062 |
| *Bdnf* | 0.01 (-0.05 – 0.07) | 0.824 | 0.005/-0.094 |
| *Ngf1* | -0.01 (-0.08 – 0.07) | 0.822 | 0.005/-0.094 |
| *Gad67* | 0.01 (-0.02 – 0.03) | 0.642 | 0.022/-0.075 |
| *Glud1* | 0.01 (-0.02 – 0.03) | 0.509 | 0.045/-0.051 |
| *Pgc1-α* | 0.01 (-0.01 – 0.03) | 0.520 | 0.043/-0.053 |
| *Sp1* | -0.01 (-0.02 – 0.01) | 0.311 | 0.102/0.012 |

**Supplemental Table 6.** Female astrocyte cultures treated with propionate (linear regression)

| **Gene** | **Estimates for Dose** | ***p-*Value** | **R/R^2^ adjusted** |
| --- | --- | --- | --- |
| *Ahr* | 0.00 (-0.01 – 0.02) | 0.829 | 0.005/-0.095 |
| *S100β* | -0.00 (-0.02 – 0.02) | 0.981 | 0.000/-0.100 |
| *Gfap* | -0.02 (-0.03 – 0.00) | 0.111 | 0.235/0.158 |
| *Ifnar* | 0.00 (-0.05 – 0.05) | 0.955 | 0.000/-0.100 |
| *Il-22* | 0.02 (0.02 – 0.06) | 0.328 | 0.096/0.005 |
| *Cyp1b1* | -0.00 (-0.02 – 0.02) | 0.902 | 0.002/-0.098 |
| *Glul* | -0.01 (-0.06 – 0.05) | 0.823 | 0.005/-0.094 |
| *Bdnf* | -0.00 (-0.06 – 0.06) | 0.944 | 0.001/-0.099 |
| *Ngf1* | -0.00 (-0.06 – 0.06) | 0.940 | 0.001/-0.099 |
| *Gad67* | 0.00 (-0.05 – 0.06) | 0.974 | 0.000/-0.100 |
| *Glud1* | -0.00 (-0.02 – 0.01) | 0.664 | 0.020/-0.078 |
| *Pgc1-α* | -0.01 (-0.03 – 0.01) | 0.379 | 0.078/-0.014 |
| *Sp1* | -0.01 (-0.04 – 0.01) | 0.229 | 0.141/0.055 |

**Supplementary Table 7:** Mean and standard error of gene expression changes after treating astrocytes with acetate

| **Acetate Dose (μM)** | | | | | | | | | |
| --- | --- | --- | --- | --- | --- | --- | --- | --- | --- |
| **Sex** | **Gene** | *0* | | *150* | | *750* | | *1500* | |
|  |  | Mean (ddct) | SE | Mean (ddct) | SE | Mean (ddct) | SE | Mean (ddct) | SE |
| **Male** | *Ahr* | 0.084 | 0.886 | 0.252 | 1.582 | 0.868 | 2.230 | 0.709 | 0.084 |
|  | *S100b* | 0.524 | 1.049 | 0.414 | 1.109 | 0.579 | 1.359 | 0.381 | 0.524 |
|  | *Gfap* | 0.372 | 1.182 | 1.074 | 1.258 | 0.549 | 2.942 | 1.236 | 0.372 |
|  | *Ifnar* | 0.519 | 0.898 | 1.069 | 1.172 | 1.193 | 1.121 | 1.051 | 0.519 |
|  | *Il-22* | 0.385 | 0.811 | 0.540 | 1.093 | 0.366 | 0.790 | 0.404 | 0.385 |
|  | *Cyp1b1* | 0.127 | 0.885 | 0.256 | 1.024 | 0.227 | 1.430 | 0.783 | 0.127 |
|  | *Glul* | 0.907 | 1.151 | 1.800 | 1.104 | 1.841 | 2.311 | 3.731 | 0.907 |
|  | *Bdnf* | 0.603 | 1.210 | 1.255 | 1.174 | 1.386 | 1.106 | 1.060 | 0.603 |
|  | *Ngf1* | 0.922 | 0.991 | 1.596 | 1.203 | 2.053 | 1.547 | 2.486 | 0.922 |
|  | *Gad67* | 0.324 | 1.252 | 0.533 | 1.600 | 1.096 | 1.541 | 0.640 | 0.324 |
|  | *Glud1* | 0.312 | 0.694 | 0.602 | 1.651 | 1.700 | 1.665 | 0.488 | 0.312 |
|  | *Pgc1-a* | 0.351 | 0.442 | 0.306 | 0.383 | 0.123 | 1.127 | 0.791 | 0.351 |
|  | *Sp1* | 0.278 | 0.883 | 0.762 | 0.616 | 0.298 | 1.002 | 0.850 | 0.278 |
| **Female** | *Ahr* | 0.180 | 0.935 | 0.223 | 1.622 | 0.657 | 1.104 | 0.173 | 0.180 |
|  | *S100b* | 0.378 | 0.648 | 0.148 | 0.997 | 0.081 | 0.926 | 0.212 | 0.378 |
|  | *Gfap* | 0.486 | 0.528 | 0.307 | 0.805 | 0.104 | 0.912 | 0.213 | 0.486 |
|  | *Ifnar* | 0.521 | 1.114 | 0.809 | 1.489 | 0.621 | 1.324 | 0.733 | 0.521 |
|  | *Il-22* | 0.456 | 1.783 | 0.556 | 2.955 | 1.741 | 1.306 | 0.382 | 0.456 |
|  | *Cyp1b1* | 0.155 | 1.128 | 0.417 | 1.377 | 0.383 | 1.055 | 0.282 | 0.155 |
|  | *Glul* | 0.425 | 1.397 | 0.856 | 1.384 | 0.664 | 0.988 | 0.546 | 0.425 |
|  | *Bdnf* | 0.446 | 1.121 | 0.695 | 1.037 | 0.422 | 0.762 | 0.353 | 0.446 |
|  | *Ngf1* | 0.822 | 1.566 | 1.406 | 1.586 | 1.341 | 1.678 | 1.523 | 0.822 |
|  | *Gad67* | 0.623 | 1.004 | 0.612 | 1.583 | 0.996 | 1.173 | 0.724 | 0.623 |
|  | *Glud1* | 0.208 | 1.364 | 0.313 | 2.224 | 0.877 | 1.492 | 0.239 | 0.208 |
|  | *Pgc1-a* | 0.363 | 0.816 | 0.247 | 1.345 | 0.505 | 0.825 | 0.296 | 0.363 |
|  | *Sp1* | 0.587 | 0.800 | 0.262 | 1.257 | 0.805 | 0.730 | 0.221 | 0.587 |

**Supplementary Table 8:** Mean and standard error of gene expression changes after treating astrocytes with butyrate

| **Butyrate Dose (μM)** | | | | | | | | | |
| --- | --- | --- | --- | --- | --- | --- | --- | --- | --- |
| **Sex** | **Gene** | *0* | | *2.5* | | *12.5* | | *25* | |
|  |  | Mean (ddct) | SE | Mean (ddct) | SE | Mean (ddct) | SE | Mean (ddct) | SE |
| **Male** | *Ahr* | 1 | 0.079 | 1.243 | 0.319 | 1.310 | 0.432 | 1.518 | 0.496 |
|  | *S100b* | 1 | 0.420 | 1.871 | 1.121 | 2.201 | 1.311 | 2.099 | 1.124 |
|  | *Gfap* | 1 | 0.277 | 1.979 | 0.662 | 1.851 | 0.610 | 1.914 | 0.906 |
|  | *Ifnar* | 1 | 0.355 | 1.139 | 0.213 | 1.139 | 0.138 | 1.180 | 0.232 |
|  | *Il-22* | 1 | 0.755 | 0.725 | 0.427 | 0.944 | 0.490 | 0.815 | 0.316 |
|  | *Cyp1b1* | 1 | 0.103 | 1.061 | 0.033 | 1.302 | 0.190 | 1.091 | 0.246 |
|  | *Glul* | 1 | 0.848 | 0.978 | 0.719 | 0.844 | 0.742 | 1.246 | 0.666 |
|  | *Bdnf* | 1 | 0.470 | 1.573 | 0.837 | 1.372 | 0.934 | 1.530 | 0.496 |
|  | *Ngf1* | 1 | 0.933 | 0.808 | 0.701 | 1.040 | 0.938 | 1.009 | 0.820 |
|  | *Gdnf* | 1 | 0.413 | 2.117 | 1.252 | 1.834 | 1.053 | 1.529 | 0.679 |
|  | *Gad67* | 1 | 0.176 | 1.081 | 0.458 | 1.483 | 0.542 | 0.905 | 0.304 |
|  | *Glud1* | 1 | 0.299 | 1.211 | 0.408 | 1.752 | 1.002 | 1.289 | 0.696 |
|  | *Pgc1-a* | 1 | 0.268 | 1.357 | 0.447 | 0.934 | 0.438 | 1.402 | 0.374 |
|  | *Sp1* | 1 | 0.193 | 1.040 | 0.190 | 0.835 | 0.338 | 1.221 | 0.315 |
| **Female** | *Ahr* | 1 | 0.073 | 1.093 | 0.188 | 1.510 | 0.347 | 1.269 | 0.256 |
|  | *S100b* | 1 | 0.457 | 1.222 | 0.533 | 1.397 | 0.697 | 1.480 | 0.841 |
|  | *Gfap* | 1 | 0.369 | 1.480 | 0.300 | 1.640 | 0.355 | 1.865 | 0.709 |
|  | *Ifnar* | 1 | 0.480 | 0.986 | 0.378 | 1.007 | 0.347 | 1.009 | 0.295 |
|  | *Il-22* | 1 | 0.684 | 0.937 | 0.582 | 0.979 | 0.568 | 0.924 | 0.835 |
|  | *Cyp1b1* | 1 | 0.055 | 0.985 | 0.129 | 0.920 | 0.032 | 1.072 | 0.184 |
|  | *Glul* | 1 | 0.498 | 1.621 | 0.308 | 1.598 | 0.239 | 2.388 | 1.021 |
|  | *Bdnf* | 1 | 0.322 | 1.239 | 0.236 | 1.422 | 0.201 | 2.071 | 0.412 |
|  | *Ngf1* | 1 | 0.849 | 1.273 | 0.914 | 1.198 | 0.780 | 1.511 | 0.802 |
|  | *Gdnf* | 1 | 0.571 | 0.780 | 0.468 | 0.873 | 0.491 | 0.904 | 0.351 |
|  | *Gad67* | 1 | 0.324 | 0.737 | 0.177 | 0.994 | 0.160 | 1.489 | 0.589 |
|  | *Glud1* | 1 | 0.152 | 1.089 | 0.094 | 1.178 | 0.281 | 0.909 | 0.197 |
|  | *Pgc1-a* | 1 | 0.275 | 1.414 | 0.352 | 1.427 | 0.252 | 2.248 | 0.607 |
|  | *Sp1* | 1 | 0.250 | 1.480 | 0.383 | 1.413 | 0.201 | 1.747 | 0.483 |

**Supplementary Table 9:** Mean and standard error of gene expression changes after treating astrocytes with propionate

| **Propionate Dose (μM)** | | | | | | | | | |
| --- | --- | --- | --- | --- | --- | --- | --- | --- | --- |
| **Sex** | **Gene** | *0* | | *3.5* | | *17.5* | | *35* | |
|  |  | Mean (ddct) | SE | Mean (ddct) | SE | Mean (ddct) | SE | Mean (ddct) | SE |
| **Male** | *Ahr* | 1 | 0.410 | 0.960 | 0.210 | 0.825 | 0.271 | 0.886 | 0.150 |
|  | *S100b* | 1 | 0.300 | 1.001 | 0.396 | 1.296 | 0.537 | 1.137 | 0.332 |
|  | *Gfap* | 1 | 0.209 | 0.837 | 0.143 | 0.592 | 0.185 | 0.582 | 0.271 |
|  | *Ifnar* | 1 | 0.420 | 0.741 | 0.303 | 0.768 | 0.342 | 0.862 | 0.594 |
|  | *Il-22* | 1 | 0.354 | 1.179 | 0.079 | 1.343 | 0.376 | 1.843 | 0.286 |
|  | *Cyp1b1* | 1 | 0.166 | 0.980 | 0.169 | 0.987 | 0.136 | 0.995 | 0.254 |
|  | *Glul* | 1 | 0.828 | 0.785 | 0.616 | 0.972 | 0.843 | 1.626 | 1.572 |
|  | *Bdnf* | 1 | 0.675 | 0.943 | 0.753 | 1.192 | 0.908 | 1.162 | 0.953 |
|  | *Ngf1* | 1 | 0.914 | 1.389 | 1.347 | 0.769 | 0.728 | 0.959 | 0.905 |
|  | *Gad67* | 1 | 0.357 | 0.734 | 0.296 | 0.871 | 0.307 | 1.104 | 0.503 |
|  | *Glud1* | 1 | 0.368 | 0.554 | 0.032 | 1.054 | 0.416 | 1.055 | 0.372 |
|  | *Pgc1-a* | 1 | 0.123 | 0.634 | 0.159 | 0.988 | 0.278 | 1.035 | 0.383 |
|  | *Sp1* | 1 | 0.092 | 0.526 | 0.059 | 0.765 | 0.136 | 0.611 | 0.093 |
| **Female** | *Ahr* | 1 | 0.106 | 1.241 | 0.202 | 1.340 | 0.225 | 1.116 | 0.161 |
|  | *S100b* | 1 | 0.245 | 1.073 | 0.299 | 1.019 | 0.311 | 1.025 | 0.369 |
|  | *Gfap* | 1 | 0.249 | 0.977 | 0.218 | 0.603 | 0.302 | 0.501 | 0.284 |
|  | *Ifnar* | 1 | 0.442 | 1.137 | 0.692 | 1.074 | 0.704 | 1.104 | 0.884 |
|  | *Il-22* | 1 | 0.212 | 0.976 | 0.466 | 1.018 | 0.364 | 1.676 | 0.940 |
|  | *Cyp1b1* | 1 | 0.211 | 1.069 | 0.300 | 0.965 | 0.275 | 0.999 | 0.306 |
|  | *Glul* | 1 | 0.590 | 1.354 | 1.037 | 1.022 | 0.709 | 0.962 | 0.713 |
|  | *Bdnf* | 1 | 0.640 | 1.320 | 0.933 | 1.068 | 0.772 | 1.079 | 0.900 |
|  | *Ngf1* | 1 | 0.835 | 0.866 | 0.714 | 1.062 | 0.964 | 0.847 | 0.771 |
|  | *Gad67* | 1 | 0.423 | 1.701 | 1.156 | 1.227 | 0.489 | 1.343 | 0.610 |
|  | *Glud1* | 1 | 0.080 | 1.125 | 0.291 | 1.013 | 0.231 | 0.940 | 0.246 |
|  | *Pgc1-a* | 1 | 0.338 | 0.907 | 0.360 | 0.742 | 0.246 | 0.649 | 0.328 |
|  | *Sp1* | 1 | 0.428 | 1.124 | 0.357 | 0.739 | 0.108 | 0.618 | 0.294 |
