# Supplementary figures and images for: Microbially-derived short-chain fatty acids impact astrocyte gene expression in a sex-specific manner
Source: Brain Behav Immun Health. 2021 Aug 6;16:100318. doi: 10.1016/j.bbih.2021.100318 (PMC8474187; doi:10.1016/j.bbih.2021.100318)

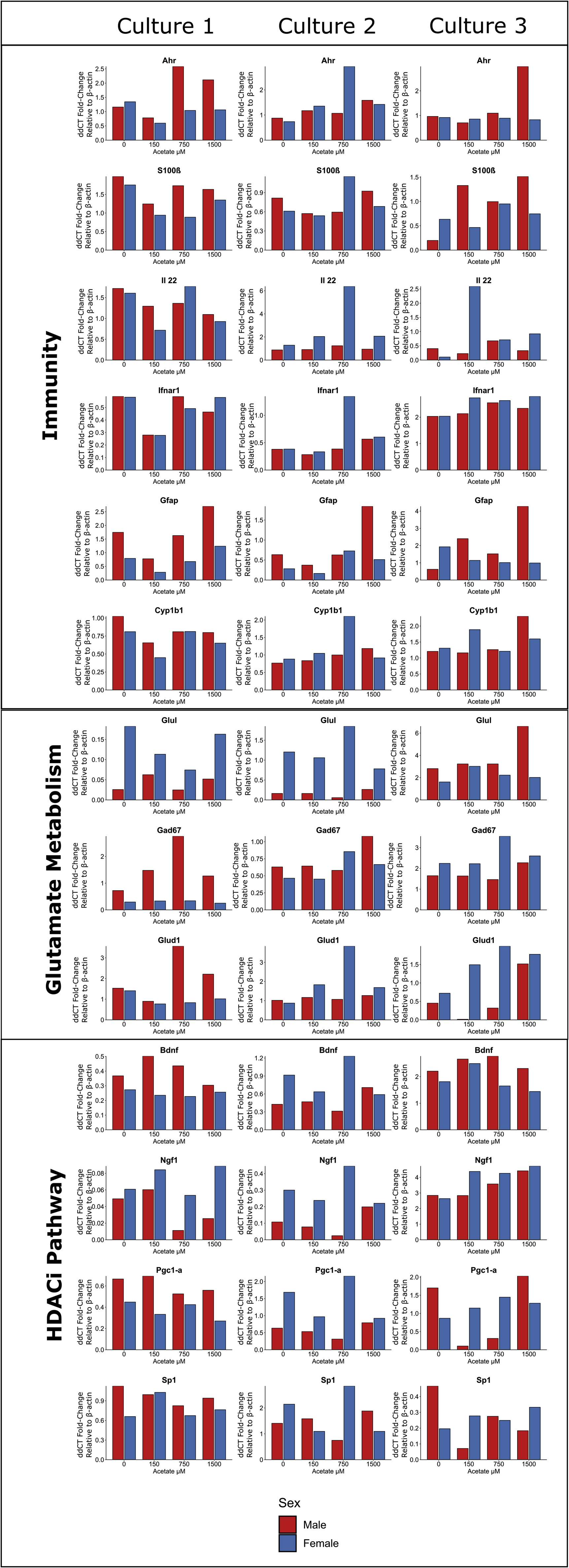

Supplement: Multimedia component 2 [file figs1.jpg]

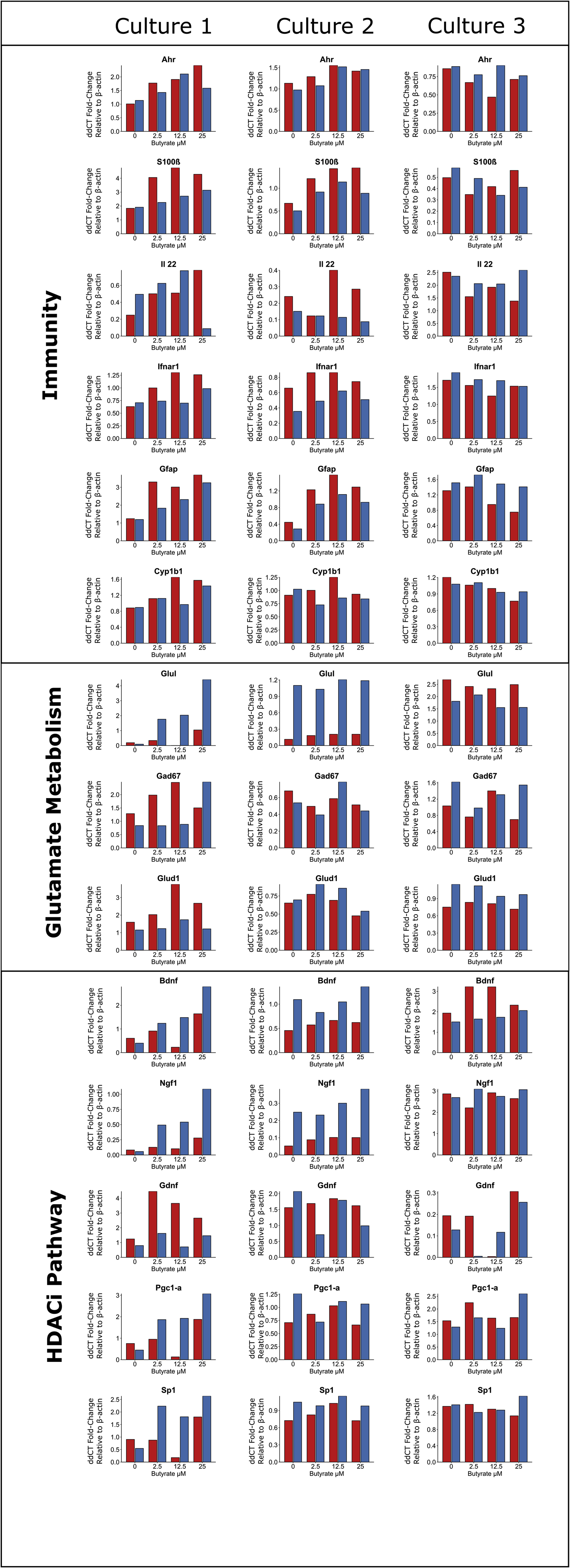

Supplement: Multimedia component 3 [file figs2.jpg]

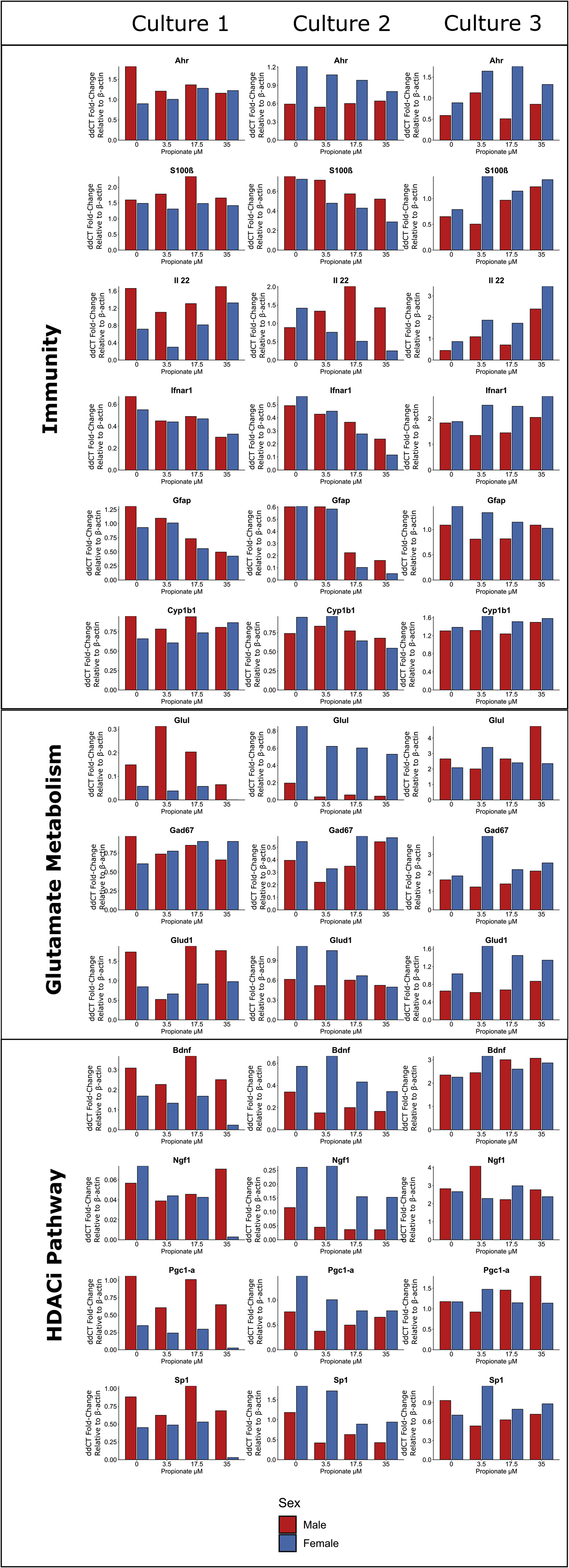

Supplement: Multimedia component 4 [file figs3.jpg]
